# Supplementary material for: Pre-intervention characteristics of the mosquito species in Benin in preparation for a randomized controlled trial assessing the efficacy of dual active-ingredient long-lasting insecticidal nets for controlling insecticide-resistant malaria vectors
Source: PLoS One. 2021 May 20;16(5):e0251742. doi: 10.1371/journal.pone.0251742 (PMC8136630; doi:10.1371/journal.pone.0251742)
Supplement: S1 Fig — (DOCX) [file pone.0251742.s001.docx]

S1 Fig: *An. gambiae* s.l. hourly biting rates in Cove (N=975 indoors, N=556 outdoors) (a), Zangnanado (N=2792 indoors, N=2134 outdoors) (b) &, and Ouinhi (N=2606 indoors, N=1744 outdoors) (c). b/p/h=bite/person/hour, the error bars indicate the confidence intervals.

a
