# Supplementary material for: Pre-intervention characteristics of the mosquito species in Benin in preparation for a randomized controlled trial assessing the efficacy of dual active-ingredient long-lasting insecticidal nets for controlling insecticide-resistant malaria vectors
Source: PLoS One. 2021 May 20;16(5):e0251742. doi: 10.1371/journal.pone.0251742 (PMC8136630; doi:10.1371/journal.pone.0251742)
Supplement: S1 Table — (DOCX) [file pone.0251742.s002.docx]

Table S1: Parous rate in *An. gambiae* s.l. in Cove, Ouinhi and Zangnanando

| Districts | Biting location | N dissected | N parous | PR (%) | 95% CI |
| --- | --- | --- | --- | --- | --- |
| Cove | Indoor | 247 | 187 | 75.2^a^ | 55.2 - 100 |
|  | Outdoor | 117 | 97 | 82.9^a^ | 53.6 - 100 |
|  |  |  |  |  |  |
| Zangnanado | Indoor | 975 | 802 | 82.2^a^ | 71.2 - 94.4 |
|  | Outdoor | 576 | 469 | 80.6^a^ | 67.4 - 95.7 |
|  |  |  |  |  |  |
| Ouinhi | Indoor | 558 | 472 | 84.6^a^ | 70.0 - 100 |
|  | Outdoor | 370 | 300 | 80.3^a^ | 63.3 - 100 |
| Study area | Indoor | 1780 | 1461 | 82.2^a^ | 74.0 - 91.0 |
|  | Outdoor | 1063 | 866 | 80.7^a^ | 70.7 - 91.8 |
|  | Total | 2843 | 2327 | 81.6 | 75.4 - 88.4 |

PR: parous rate, CI: confidence interval
