# Supplementary material for: Pre-intervention characteristics of the mosquito species in Benin in preparation for a randomized controlled trial assessing the efficacy of dual active-ingredient long-lasting insecticidal nets for controlling insecticide-resistant malaria vectors
Source: PLoS One. 2021 May 20;16(5):e0251742. doi: 10.1371/journal.pone.0251742 (PMC8136630; doi:10.1371/journal.pone.0251742)
Supplement: S2 Table — (DOCX) [file pone.0251742.s003.docx]

Table S2: Allelic frequencies of the L1014F *kdr* mutation in *An. gambiae* s.s and *An. coluzzii* s.s. collected using HLCs.

|  |  |  |  |  |  |  |  |
| --- | --- | --- | --- | --- | --- | --- | --- |
|  |  |  | Genotypes | | |  |  |
| Districts | Molecular species | N Tested | RR | RS | SS | F (L1014F *kdr*) | 95% CI |
| Cove | *Anopheles coluzzii* | 126 | 90 | 32 | 4 | 84.1^a^ | 79.0-88.4 |
|  | *Anopheles gambiae s.s.* | 116 | 96 | 19 | 1 | 90.9^a^ | 86.4-94.3 |
|  |  |  |  |  |  |  |  |
| Zangnanando | *Anopheles coluzzii* | 341 | 245 | 79 | 17 | 83.4^a^ | 80.4-86.1 |
|  | *Anopheles gambiae s.s.* | 636 | 525 | 101 | 10 | 90.5^b^ | 88.7- 92.0 |
|  |  |  |  |  |  |  |  |
| Ouinhi | *Anopheles coluzzii* | 501 | 374 | 103 | 24 | 84.9^a^ | 82.5-87.1 |
|  | *Anopheles gambiae s.s.* | 77 | 54 | 19 | 4 | 82.5^a^ | 75.5- 88.1 |
| Total | *Anopheles coluzzii* | 968 | 709 | 214 | 45 | 84.3^a^ | 82.5-85.9 |
|  | *Anopheles gambiae s.s.* | 829 | 675 | 139 | 15 | 89.8^b^ | 88.2-91.2 |

F: frequency, CI: confidence interval, a, b: Values of F(L1014F *kdr*) with different superscripts within a same district are significantly different
