# Supplementary material for: Pre-intervention characteristics of the mosquito species in Benin in preparation for a randomized controlled trial assessing the efficacy of dual active-ingredient long-lasting insecticidal nets for controlling insecticide-resistant malaria vectors
Source: PLoS One. 2021 May 20;16(5):e0251742. doi: 10.1371/journal.pone.0251742 (PMC8136630; doi:10.1371/journal.pone.0251742)
Supplement: Attachment — Submitted filename: Report PONE-D-21-02133_reviewer.pdf [file pone.0251742.s004.pdf]

Reviewer's report

Title: Pre-intervention characteristics of the mosquito species in Benin in preparation for a Randomized Controlled Trial assessing the efficacy of dual active-ingredient long- lasting insecticidal nets for controlling insecticide-resistant malaria vectors

General comments

This study of **Pre-intervention characteristics of the mosquito species in Benin in preparation for a Randomized Controlled Trial assessing the efficacy of dual active-ingredient long- lasting insecticidal nets for controlling insecticide-resistant malaria vectors** represents an important topic related to the bionomics of malaria vectors and insecticide resistance**.**

To study the entomological indicators in malaria vectors, human landing catch method is used. Prior this, informed consent was obtained from each collector. Moreover, the study protocol has been approved by the Benin national ethics committee for health research.

I could recommend the current version of this paper for publication after the very minor additive data below.

Version: 1

Date: 14 **April** 2021

Reviewer number: 1

Minor revisions

- In 178 to 179, Indicate the geographical coordinates of each district (Covè, Ouinhi and, Zagnanando)   in terms of longitude and latitude.
- Indicate the North symbol/direction (N) of the compass on figure 1 legend (Map of study area)
- Line 177 to 188: Authors should align the font of the paragraph similar to the rest text.
- Authors could choose to underlined each paragraph titles or not. If so, then they need to do throughout the document.
- Line 242 (Data management and analysis): Include in the table, the formulas for indoor and outdoor rates respectively.
- HBR, SR and EIR in An. gambiae s.l. in Cove, Ouinhi and Zangnanado were reported. Have authors also stratified EIR data also according to the An. gambiae species (i.e EIR in An. coluzzii and An. gambiae s.s respectively)? It will be interesting if available to include in the manuscript.
- Lines 304/305; 383/384: Because, Culex and Aedes represent the major mosquito's populations from the study area, authors should also discuss the strategies to reduce their effect on human, in the perspective of future LLINs trial, given the noising effect of Culex, and Aedes effect on human through dengue disease as described in many other countries.
- Line 327/328: What the error bars mean on Figure 3? Is it confidence intervals (CI) or Standard error of means (SEM)? Please add in figure legend.
- Line 367/368: What the error bars mean on Figure 4? Is it confidence intervals (CI) or Standard error of means (SEM)? Please add in figure legend.
- Line 369/370: What the error bars mean on Figure 5? Is it confidence intervals (CI) or Standard error of means (SEM)? Please add in figure legend
- Line 580 to 581: Please check for line spacing and homogenise accordingly

Level of interest: Statistical analysis added using Stata

Level of interest: An article of importance in its field

Quality of written English: Acceptable
